# Comparative Evaluation of HVT-IBD Vector, Immune Complex, and Live IBD Vaccines against vvIBDV in Commercial Broiler Chickens with High Maternally Derived Antibodies

**DOI:** 10.3390/ani9030072

**Published:** 2019-02-26

**Authors:** Mahmoud E. Sedeik, Nahed A. El-shall, Ashraf M. Awad, Mohamed E. Abd El-Hack, Abdullah N. Alowaimer, Ayman A. Swelum

**Affiliations:** 1 Department of Poultry and Fish Diseases, Faculty of Veterinary Medicine, Alexandria University, Edfina, Elbehira 22758, Egypt; seddeeklab@yahoo.com (M.E.S.); nahed.elshall@gmail.com (N.A.E.); dr.ashrafawad@gmail.com (A.M.A.); 2 Department of Poultry, Faculty of Agriculture, Zagazig University, Zagazig 44511, Egypt; 3 Department of Animal Production, College of Food and Agriculture Sciences, King Saud University, P.O. Box 2460, Riyadh 11451, Saudi Arabia; aowaimer@ksu.edu.sa; 4 Department of Theriogenology, Faculty of Veterinary Medicine, Zagazig University, Zagazig 44519, Egypt

**Keywords:** infectious bursal disease, vvIBDV, HVT-IBD vector, immune complex, ELISA

## Abstract

**Simple Summary:**

Vaccination is the main method to control infectious bursal disease (IBD) in commercial broilers worldwide. The main obstacle to the vaccination process is maternally derived antibodies; thus, new generations of vaccines such as vector and immune complex vaccines have been developed. The efficacies of new and classical vaccines were compared to those of vvIBDV in the presence of high levels of maternally derived antibodies. The best results were obtained when using the vector IBD vaccine followed by the immune-complex vaccine, and the use of killed along with the live intermediate vaccine in terms of mortality, feed conversion ratio, bursal and spleen index, bursal lesion score, and serology.

**Abstract:**

Infectious bursal disease (IBD) causes increased mortality and severe immunosuppression in commercial chickens. Currently, vaccination mainly used to control IBD. In this study, Group A (n = 30) received the HVT-IBD vector vaccine *(Vaxxitek^®^)* s/c and Group B (n = 30) received the immune complex vaccine *(Bursa-Plex^®^)* s/c at 1 day of age. Group C (n = 30) received a single dose of intermediate plus vaccine *(228E)* through the eye-drop route at 14 days of age. Group D (n = 30) was vaccinated twice with the intermediate vaccine *(D78)* at 12 and 22 days of age by eye-drop. Group E (n = 30) had the same treatment as group D along with the IBD killed vaccine (Nobilis G^®^) at 5 days of age. The PC (n = 20) and NC (n = 20) groups were non IBD vaccinated birds either challenged or not with vvIBDV, respectively; 20 chicks from each group were challenged with vvIBDV at 4 weeks of age. Based on clinical signs, postmortem gross lesions, histopathological changes, mortality rate, feed conversion rate, serology, bursal and spleen indices, the HVT-IBD vector vaccine administered was found to be safer and provided better protection against the vvIBDV challenge. The use of a killed IBD vaccine at an earlier age in broilers strengthened the protection induced by double doses of intermediate vaccines in broilers with high maternally derived antibodies against the vvIBDV challenge.

## 1. Introduction

Infectious bursal disease (IBD) or Gumboro disease is an acute and highly contagious disease affecting young chickens [1], caused by the infectious bursal disease virus (IBDV). The virus is a member of the Birnaviridae family, having a bi-segmented double stranded RNA enclosed within a non-enveloped capsid [2]. The first report of IBD was in 1962 in the USA, then it was firstly reported in Egypt by El-Sergany [3], from that time it continues to cause drastic economic losses due to its high mortality rate, which could reach up to 30% and 60% in broilers and layers, respectively, with an ability to impair growth, and cause immunosuppression [4,5] as IBDV destroys the precursor antibody-producing cells in the bursa of Fabricius, particularly B-lymphocytes, inducing bursal atrophy [6].

The IBD virus is highly resistant to adverse environmental conditions resulting in a long persistence in poultry farms; thus, effective vaccination along with strict hygiene management is essential for the prevention of IBD [7]. However, IBDV is evolving quickly in the field [8], resulting in the emergence of antigenic variants of IBDV in the early 1980s [9] and very virulent IBDV (vvIBDV) strains in the late 1980s [10]. Egypt is not far from what occurs, as variant strains of IBDV was recorded [11,12], but the most prevalent IBDV strain in the field is vvIBDV [12,13,14,15] and thus, the situation with IBDV is becoming more complex [16] and the development of the IBD virus’s antigenicity and virulence has made the control of IBDV by vaccination more challenging [17].

Conventional attenuated live vaccines containing classical or variant virus strains and killed vaccines are commercially available and most commonly used all over the world [18]. According to the degree of attenuation, live vaccines are classified as mild, intermediated, intermediate plus, and hot IBD vaccines [19], where mild and intermediate vaccines are safer than the intermediate plus and hot vaccines as they cause less bursal damage; but are easily neutralized by high levels of maternally derived antibodies (MDAbs).

With the advancement of technology, next-generation vaccines have been developed with the advantage of overcoming MDAbs and are commercially available in the market such as the IBD vector vaccine using turkey herpes virus (HVT) as a vector for the IBDV viral protein 2 *(VP2)* gene [20], and the Immune-complex vaccine that is a mixture of the intermediate plus strain with antibodies, which is taken up by macrophages till the MDAbs have been dropped [21]. 

In Egypt, there are different vaccination programs used to control the IBDV, but many farmers do not follow the recommendation of measuring MDAbs before the application of a vaccine. Thus, this trial was conducted to simulate field conditions and evaluate the protection efficacy of some different vaccination programs against vvIBDV in commercial broiler chickens.

## 2. Material and Methods

### 2.1. IBDV Vaccines

A commercial vector vaccine HVT-IBD *(Vaxxitek^®^)* (Merial S.A.S., USA), that was generated by inserting an IBDV *VP2* gene (cloned from Faragher 52/70 IBDV strain) into the HVT genome, was subcutaneously (s/c) injected in one-day-old chicks at the hatchery. An Immune-complex vaccine *(Bursa-Plex^®^)* (Zoetis Inc, Parsippany NJ, New York, USA) was given s/c to one-day-old chicks at the hatchery. Two classic live vaccines, intermediate plus vaccine *“Nobilis GUMBORO 228E^®^” (MSD Inc, Kenilworth, NJ, USA)* and intermediate *vaccine “Nobilis GUMBORO D78^®^”* (MSD Inc, Kenilworth, NJ, USA), were administered through eye drops, in addition to the inactivated IBD vaccine *“Nobilis G^®^”* (MSD Inc, Kenilworth, NJ, USA) by s/c injection. The four vaccines used in this study simulate the field conditions of various programs operational in Egypt that address the IBDV challenge.

### 2.2. IBD Virus

A local field vvIBDV **“**Egypt-Behira-29-2017” isolated from 25-day-old SASO chicks with a mortality rate of 6.7% [22] was characterized through sequencing of only VP2 and submitted to the gene bank under accession number MG599731, which was used for the challenge. It was tit rated by inoculation in 10-day-old specific pathogen free-embryonated chicken eggs (SPF-ECEs) to calculate embryo infective dose 50 (EID_50_) according to Reed et al. [23] and used with a challenge dose of 10^3.5^ EID_50_/ml (100 µL/bird: 50 µL by the nasal route and 50 µL using eye drops) [24] at 28 days of age.

### 2.3. Chickens and Vaccination Programs

This trial was performed strictly according to the recommendations and guidelines of the committee on the ethics of animal experiments of Alexandria University, Egypt (ALEX-47019). All efforts were made to minimize suffering. One hundred and ninety-one-day-old commercial broiler chicks (Avian 48), were obtained from a local hatchery (El-kanana, Tanta, Egypt), and reared in clean well-ventilated floor pens with 10 cm depth fresh wood shavings litter at the poultry diseases clinic, Faculty of Veterinary Medicine, Alexandria University. The chicks were received at 33 °C, then the temperature decreased by 2 degrees every week. Feed and water were supplied *ad libitum* where the feed types were a starter (crumbles) for 1–14 days with 23% crude protein and metabolizable energy (ME) 3008 Kcal/kg diet, grower (pellets) for 15–28 days with 21% crude protein and ME 3080 Kcal/kg diet and finisher (pellets) feed for 29–35 days with 19% crude protein and ME 3190 Kcal/kg diet.

The birds were distributed into seven groups (A–E, positive control (PC), and negative control (NC). Group A (n = 30) and Group B (n = 30) were vaccinated with the HVT-IBD vector vaccine *(Vaxxitek^®^)* s/c and the immune-complex vaccine *(Bursa-Plex^®^)* s/c, respectively, when one day old at the hatchery. Group C (n = 30) received a single dose of the intermediate plus vaccine *(228E)* by the eye drop route according to Hair-Bejo et al. [25]; Moraes et al. [26], and El-mahdy et al. [27] at 14 days of age. Group D (n = 30) was vaccinated twice with the intermediate vaccine *(D78)* at 12 and 22 days of age using eye drops. Group E (n = 30) was administered the same treatment as group D, and in addition it was injected with the IBD killed vaccine *(Nobilis G^®^)* at 5 days of age. PC (n = 20) and NC (n = 20) groups were non-IBD vaccinated birds either challenged or not with the vvIBDV, respectively. The challenge was performed on 20 chicks from each group with vvIBDV at 4 weeks of age and the remaining 10 chicks in the groups (A–E) were isolated without challenge for further serological and histopathological examination and measuring of organ indexes to study the impact of different IBDV vaccines on chickens.

Serum samples were collected from additional ten one-day-old chicks to determine the MDAbs titer for IBDV. Then, throughout the experiment, ten chicks were randomly selected from each group for blood sampling from the wing vein at the 3rd, 4th, and 5th weeks of age to obtain sera in addition to serum samples from the ten non-challenged chicks of each group at 35 days of age. Wing tags were used to individually identify the chicks to monitor their body weight. 

Birds of all groups received a full dose of Newcastle (ND) and Infectious Bronchitis (IB) diseases live attenuated vaccines *(Nobilis MA5+Clone 30^®^)(MSD)* at 7 and 18 days of age via the eye drop route as well as the ND inactivated vaccine *(Nobilis ND^®^)* (MSD Inc, Kenilworth, NJ, USA) at 10 days of age through the s/c injection in the neck region. 

### 2.4. Clinical Signs, Mortality, and Postmortem Lesions

The birds were observed daily for any clinical signs during the experimental period. Dead birds were examined for macroscopic lesions. At the end of the experiment, (7 days post challenge (dpch)), a postmortem examination was performed on all surviving birds.

### 2.5. Bursa to Body Weight Index, Spleen Index, and Histopathology

At 5 weeks of age (1-week post challenge), 6/20 birds from challenged groups either vaccinated (A–E) or non-vaccinated (PC), 6/10 non challenged birds for groups (A–E) plus 6/20 non challenged birds for NC group were selected randomly, weighed (using electronic price computing scale, serial No. 201, China) and taken for a postmortem examination. Bursae and Spleen were collected and weighed individually (using digital balance, serial No. J8094650, Japan) to calculate the bursa/body weight ratio = bursa weight (gram)/bird weight (gram) × 1000 [28] and bursa/body weight index = (Bursa/body weight ratio of each bird)/(Mean Bursa/body weight ratio of uninfected control birds) [29] and the spleen index was calculated as follows: Organ index = organ weight (gram)/body weight (gram) × 1000 [30].

For histopathological examination, bursae and spleen were fixed in 10% formalin, embedded in paraffin, and sectioned using a microtome into slices of 4–6 μm thickness and then stained with Hematoxylin and Eosin (H&E) stains. The Bursa lesion score was determined and compared between the experimental groups across a range of 0 to 4 according to the percentage of lymphoid necrosis and/or lymphocytic depletion of the lymphoid follicles per field, as following: 1 = 1–25%, 2 = 26–50%, 3 = 51–75 %, 4 = 76–100% of follicles showing lymphocytic depletion and/or necrosis [31].

### 2.6. Serology

Blood samples were collected in sterile tubes and left to clot in a sloping position at 37 °C for one hour. This was followed by overnight refrigeration, followed by centrifugation at 3000 rpm for 15 min to separate the sera and stored at −20 °C until use. Serological titration of IBD-antibodies was performed using commercial indirect classical ELISA kits (ID Vet, France). According to the manufacturer’s instructions, IBD immune status was considered negative if ELISA titer is less than 875. 

### 2.7. Feed Conversion Ratio (FCR)

FCR was calculated during the period of the challenge (4th to 5th week of age), as feed conversion ratio per bird = (Total feed consumption in a pen) ÷ (Weight gain of surviving birds + weight gain of dead birds in the same pen) [32].

### 2.8. Statistical Analysis

Group responses for the evaluated parameters were analyzed and compared using one-way analysis of variance (ANOVA) where the significance level was set at *p* ≤ 0.05. The statistical analyses were performed using the statistical package [33].

## 3. Results

### 3.1. Clinical Protection (Clinical Signs And Mortality)

No clinical signs or mortality were recorded in the experimental groups, either vaccinated against IBD or not, till the age of the challenge (4 weeks); the same observation was recorded for the negative control birds till the end of the experiment (5 weeks). Huddling together, depression, ruffling feathers, anorexia, whitish diarrhea, and soiled vents were observed among the PC birds beginning from the second dpch and continued to the seventh dpch. Three vaccinated and challenged groups (A, B, and E) did not exhibit clinical signs or mortality during this period; while group C and D showed anorexia and seven of 20 birds in group D exhibited depression plus ruffling of feathers. Four chicks (20 %) in the non-vaccinated group (PC) died on the second and fifth dpch and 10% mortality occurred in groups C (at second dpch) and D (at sixth dpch) (Table 1). 

### 3.2. Feed Conversion Ratio (FCR) for 7 Days Following vviBDV Challenge

The challenge with vvIBDV resulted in significantly (*p* < 0.05) poor feed conversion ratio in non-vaccinated birds (PC) (2.41± 0.42) compared to that in the non-challenged group (NC) (1.54 ± 0.11) at seven days post challenge. On the other hand, FCR of vaccinated groups after the vvIBDV challenge was the best in groups A and B and it did not differ significantly (*p* > 0.05) from the NC group, while there was no significant difference of FCR in groups C, D, and E compared to that of the PC group (*p* > 0.05) (Table 1).

### 3.3. Pathology and Histopathology

Dead birds showed postmortem lesions typical for IBD, like hemorrhage of the thigh and/or pectoral muscles, bursa covered with gelatinous exudate, and an enlarged spleen. All birds that survived were taken for a postmortem examination at the seventh dpch. PC birds showed hemorrhagic bursa (9/16 birds), bursa containing necrotic material (4/16), hemorrhage at the proventriculus-gizzard junction (10/16), liver congestion (13/16), nephritis (10/16), enlarged spleen (9/16), and dehydrated carcasses (7/16). None of these lesions were observed on the vaccinated groups except for hemorrhagic bursa that were recorded in group A (2/20 birds), C (6/18), and D (5/18); and dehydrated carcasses were observed in group C (2/18). Non-vaccinated, non-challenged birds (NC) did not show any macroscopic lesions.

All vaccines used in this study decreased the bursal to body weight index significantly (*p* < 0.05) in non-challenged birds compared to non-vaccinated non challenged group (NC), except to that of group A (vector IBD vaccine) (*p* > 0.05) with the lowest value of the bursal index (0.35 ± 0.06) was in group C (228E vaccine). The vvIBDV challenge resulted in a decrease in the bursal index at the seventh dpch in non-vaccinated birds (PC) and vaccinated ones, irrespective of the vaccination program used (*p* < 0.05) (Table 2).

**Figure 1:**
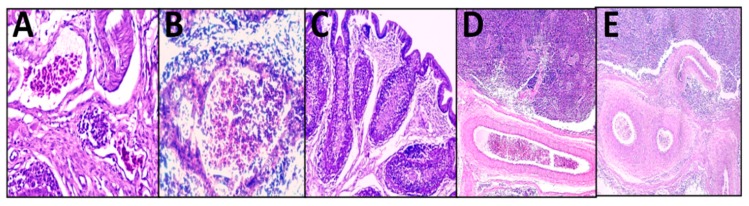
Histopathological lesions of bursae of fabricius (**A–C**) and spleen (D&E) (H&E): (**A**) Mild congestion of blood vessels (H&E × 400). (**B**) Interfollicular heterophils and macrophages aggregation (H&E × 400). (**C**) Interfollicular edema and necrosis of lymphocytic follicles replaced by heterophils and macrophages (H&E × 100). (**D**) Multifocal depletion of lymphocytes (H&E × 50). (**E**) Thickening of the wall of blood vessels (H&E × 100).

No histopathological lesions (either lymphocytic depletion and/or necrosis) were observed in group A similar to the NC group; but the other vaccines caused microscopic lesions that were detected at 35 days of age, with the highest score (1.83 ± 0.17) observed in three out of five examined birds of group E and the lowest average bursal lesion score (0.33 ± 0.33) in group D. vvIBDV infection at four weeks of age caused significant increase of the average bursal lesion score (3.17 ± 0.17) at seventh dpch in the PC group (*p* < 0.5) while group A had the lowest average score at seventh dpch (0.83 ± 0.17) (*p* < 0.05); however, this challenge did not affect the bursa histologically among the other vaccination programs (groups B, C, D, and E) (*p* > 0.05) (Figure 1 and Table 2). 

The spleen index was not significantly affected by the various vaccines against IBDV (*p* > 0.05) in comparison with that of the NC birds at 35 days of age. Nevertheless, the vvIBDV challenge at four weeks of age increased the spleen index (1.31 ± 0.32) significantly in the PC group (*p* < 0.05) in comparison to that in the other groups except group A (Table 2). Histopathological examination of spleen at seven days post challenge with vvIBDV revealed only diffused lymphocytic depletion and coagulative necrosis in the PC group, congested blood vessels with thickening of the walls in group A, and proliferation of sheathed capillaries in group E (Figure 1). 

### 3.4. Humoral Immune Response: 

**Figure 2:**
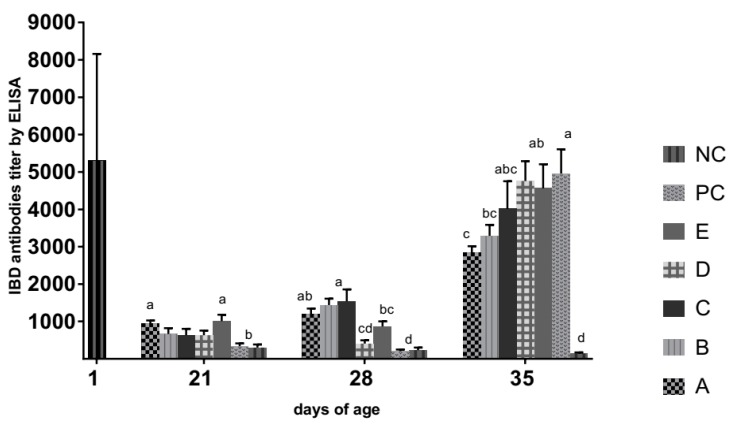
IBDv antibody titers with standard deviation in commercial broilers vaccinated with different IBD vaccines. (A): Vaccinated with HVT-IBD vector vaccine at hatchery. (B): Vaccinated with immune complex at hatchery. (C): Received intermediate plus (228E) at 14 days of age by eye drop. (D): Received two doses of intermediate (D78) at 12 and 22 days of age by eye drop. (E): Like group D but inoculated also with the IBD killed vaccine s/c at five days of age. (PC &NC): Positive and negative control birds did not receive any IBD vaccines but challenged at 28 days of age with vvIBDv (PC) and saline (NC) by oculo-nasal route. Different superscript letters on the same day indicate significant differences between groups (*p* < 0.05). IBD immune status was considered negative if ELISA titer less than 875 according to commercial indirect classical ELISA kits (ID Vet, France) manufacturer’s instructions.

An average of (5301 ± 2856) maternally derived antibodies (MDAbs) to IBDV were detected by ELISA in the one-day-old chicks and declined on non-vaccinated groups throughout the experimental period. According to ELISA manufacturer’s instructions, the IBDV immune status would be considered negative if the ELISA titer was less than 875, thus it was considered negative beginning from the third week of age in the non-vaccinated groups where MDAbs titer was 339 ± 74.5 and 302 ± 81.2 in the PC and NC groups, respectively that became 202 ± 48.8 and 231±71.9, respectively at the fourth week of age (day of challenge) (Figure 2).

Although, the IBDV vectored vaccine (group A) and two doses of live intermediate vaccine plus killed IBDV vaccine (group E) induced a comparable increase in IBDV antibodies at 21 days compared to the immune-complex (group B) and intermediate plus (group C) vaccines (*p* > 0.05), the reverse situation was observed at 28 days, where the immune-complex and intermediate plus vaccine groups showed significantly higher antibody levels than group E (*p* < 0.05) and comparable levels of antibodies (*p* > 0.05) to the IBD vectored vaccine group (Figure 2). 

Birds of group D that received two doses of intermediate IBDV vaccine had slightly lower IBDV antibodies than non-vaccinated control birds (*p* > 0.05) till the age of the challenge (28 days) (Figure 2). At one-week post challenge with vvIBDV, the non-vaccinated (PC) birds seroconverted significantly (*p* < 0.05) unlike birds of the NC, A, and B groups (Figure 2).

**Figure 3:**
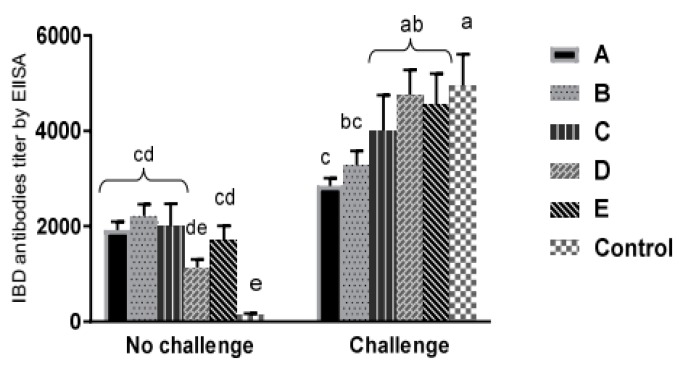
IBDV antibody titers with standard deviation in commercial broilers at 35 days of age in challenged (seven days post challenge) and non-challenged subgroups. (A): Vaccinated with HVT-IBD vector vaccine at hatchery. (B): Vaccinated with immune complex at hatchery. (C): Received intermediate plus (228E) at 14 days of age by eye drop. (D): Received two doses of intermediate (D78) at 12 and 22 days of age by eye drop. (E): Like group D but inoculated also with IBD killed vaccine s/c at five days of age. (Control): Did not receive any IBD vaccines either challenged at 28 days of age with vvIBDV or with saline (non-challenged) by oculo-nasal route. Different superscript letters indicate significant differences between groups (*p* < 0.05). IBD immune status was considered negative if ELISA titer was less than 875 according to commercial indirect classical ELISA kits (ID. Vet, France) manufacturer’s instructions.

One week post the vvIBDV challenge, an observed seroconversion occurred significantly (*p* <0.05) in all challenged groups except groups A, B, and C that had a higher antibody titer a week earlier but it was not significant (*p* > 0.05) (Figure 3).

## 4. Discussion

There are various vaccination programs for IBD prevention that differ in the timing and route of administration, frequency, vaccine strain, and vaccine interference by MDAbs. The half-life of the MDAbs and their homogeneity or heterogeneity are essential to determine the optimal time of vaccination [35]. However, many farmers apply different IBD vaccines, especially live types, without determining the MDAbs titers; therefore, in this experiment the efficacy of HVT-IBD vector, immune-complex, and live IBD vaccines, either intermediate or intermediate plus, were compared across different vaccination programs to overcome the IBD challenge in commercial broilers that had high IBD MDAbs level. 

To evaluate the efficacy of IBD vaccines, several parameters have been used such as clinical signs, mortality rate, postmortem gross lesions, IBD antibody titer, bursal index, and feed conversion ratio. The differences that were observed between the vvIBDV challenged and unchallenged birds regarding death, clinical signs, and macroscopic lesions proved that our experimental model was valid when MDAbs were high in chicks one-day post hatch; and by following the waning of them on non-vaccinated groups, they decreased to negative on the day of the challenge. 

No clinical signs, postmortem gross lesions, or mortalities were observed in all vaccinated groups till the challenge (four weeks of age), but regarding the immune suppressive effect of these vaccines, HVT-IBD vector vaccine was the only vaccine that did not cause bursal atrophy and microscopic lesions in the bursa, while the other groups that received the intermediate, intermediate plus, or immune-complex vaccines had atrophied bursae with indices below 0.7 [36] and significant increase in the microscopic bursal lesion score (*p* < 0.05) compared to those of the non-vaccinated birds. These results confirm the findings of Kumar et al. [37] that live IBD vaccines, either intermediate or intermediate plus, have a destructive effect with various degrees on the bursa, inducing transient immune suppression while HVT-IBD vector vaccine contains only VP2 that expressed on the HVT genome, thus, there is no replication of the vaccine on bursa of fabricious. 

Intermediate plus vaccines contain more pathogenic strains causing more severe lesions in the bursa than intermediate vaccines [38]; however, the bursal index did not differ significantly between groups C, D, and E, which may be attributed to the use of double doses of intermediate vaccine for groups D and E at 12 and 22 days of age. Although Haddad et al. [39] and Phatak [40] suggested that the duration of recovery may differ between IBD vaccine strains, no recovery was observed in this study as the immune suppression effect was determined at 35 days of age (age of marketing of broilers). Although the selected vaccines, except for the immune-complex, affected the spleen index negatively, this effect was not significant (*p* > 0.05), indicating adequate spleen development in the different vaccination groups, which is in agreement with the observations of Paul et al. [41].

Broiler chicks hatched with high MDAbs (5301 ± 2856) according to Le Gros et al. [42], who classified the MDAbs at one day post hatching into three groups: (<3000) Low, (3000–5000) intermediate and (>5000) high group. These MDAbs declined to reach 320 ± 26.16 in the non-vaccinated groups at 21 days of age as MDAbs level on chicks usually wane after 7–14 days or within 15–20 days post hatching [43,44], respectively. Seroconversion was significant following vaccination with HVT-IBD vector vaccine at three weeks of age than non-vaccinated groups, but in comparison with other types of vaccines, it was not significantly different and the same occurred at four weeks of age, except with group D Although this vaccine is able to produce an immune response in presence of high MDAbs according to Kenezevic et al. [45] and Berg [46], the results of IBDV antibody titers by classical ELISA kits confirmed what was reported by Chang et al. [47] and Chang et al. [48] that DNA vaccines induce low antibody ELISA titer that may be undetectable even after the IBDV challenge. Actually, the significant minimal seroconversion was recorded after the vvIBDV challenge on the HVT-IBD vector group (similar results were reported by Lemiere et al. [49]), which may be explained by antibody titer that might be high enough to neutralize part of the challenged virus and prevent its replication, thus the classical ELISA kit used for detection of antibodies may not be a good estimator of the antibodies generated by the HVT-VP2 recombinant vaccine. 

The immunocomplex vaccine resulted in poor immune response as the IBD ELISA titer was negative (663 ± 153.7) at three weeks of age (according to the ELISA Kit manufacturer’s protocol), which may be explained by the virus in the vaccine being still bound by some virus neutralizing factor resulting in poor active immunity [50]; but they increased significantly at 28 days of age (1442 ± 172). The immune response to the intermediate plus (228E) vaccine was considered negative at 21 days of age but it became significantly high after two weeks of its application (28 days of age), while Wyeth and Chettle [51] reported that seroconversion to intermediate plus vaccines in commercial broilers with a high level of MDAbs could be expected at 18 days after vaccination. 

Although Kenezevic et al. [45] reported that application of live vaccines to chickens with high levels of MDAbs failed to produce primary immune response but re-vaccination stimulated immune response, two doses of intermediate live vaccine D78 resulted in negative IBD ELISA titer till 28 days of age (day of challenge) and confirmed that the intermediate vaccines could not overcome the high MDAbs [52,53]. Nevertheless, the use of killed IBD vaccine at five days of age with two doses of intermediate D78 provoked significant immune response beginning from 21 days of age more than two doses of live vaccine on group D. It may be attributed to the fact that killed vaccines are more immunogenic, inducing humoral immunity that may neutralize part of the virus replication and improve clinical protection confirming the results of Sultan [54] that chicks need to receive live and killed vaccines to induce an immediate immune response and to maintain the high antibody titers. Moreover, Rashid [55] mentioned that killed vaccines have been used occasionally in chicks as young as 10 days old to overcome virulent viruses. 

Challenge of chickens with vvIBDV at 28-day-old age resulted in seroconversion that was reflected by higher ELISA antibody titer in the challenged groups than in the non-challenged ones. 

Although group C showed high antibody titer at 28 days of age (1542 ± 318), this group had 10% mortality, and group E that induced low antibody at 28 days (867 ± 135.5) had no mortality post the vvIBDV challenge. This may be because the ELISA antibody titer was not the sole indicator of the ability of birds to resist the vvIBDV challenge. 

Protection percentage was 100 % (deaths, clinical signs, and macroscopic lesions) in groups B (Immune-complex) and E (2 doses of intermediate and one dose of killed) while group A (HVT-IBD vector) had 100% protection against deaths and clinical signs but had some gross lesions represented on the hemorrhagic bursa. Groups C (intermediate plus) and D (2 doses of intermediate) had 10% mortality and some clinical signs and gross lesions but in a degree lighter than that occurred in the non-vaccinated one (PC). These results confirm previous reports which revealed that the severity of IBDV could be minimized by numerous vaccination programs [56,57,58].

The bursal index results showed that the bursae were atrophied in all vaccinated groups post challenge with indices below 0.7 [31], which indicated that none of these vaccines could prevent damage of the bursa of Fabricius; similar results were reported by Chansiripornchai [44] for the immune-complex and intermediate plus vaccines. Nevertheless, the minimum degree of the atrophy was recorded in group A that had the least antibody titer post challenge and this might be explained by Rautenschlein [34] who reported that the induction of humoral immunity clearly correlated with the induction of bursal lesions and IBDV replication. Similarly, group A had the lowest histopathological lesion score of bursa post challenge. All of these reflect the superiority of the vaccine used in this group in protection against the vvIBDV challenge; similar findings were reported by Le Gros et al. [42], Sultan et al. [54], and Rashid [55].

As the authors in reference [44] mentioned, the pathogenicity of IBDV was distributed to non-bursa lymphoid organs such as the spleen. The vvIBDV challenge significantly increased the spleen index at seven days post challenge in non-vaccinated birds; similar results were reported by Suliman [57]. Additionally, it induced diffuse lymphocytic depletion and coagulative necrosis. Different vaccination programs succeeded in decreasing the spleen index significantly compared to that of the PC group at seven days post challenge and prevented lymphocytic depletion and coagulative necrosis.

Independence of FCR was recorded in all groups during the challenge period (seven days); groups A and B showed the best FCR and similar results were described by Rashid [55].

Various field and experimental studies have focused on the importance of determining the optimal vaccination time, which is based on the half-life and variation of the MDAbs and the ability of the vaccinal strain to break through theMDAbs [35,36]. The results of live intermediate and intermediate plus vaccines used on groups C and D, respectively, confirmed these studies, thus using the Deventer formula [58] is essential to calculate the optimal vaccination time to avoid delayed or prevented immune response and subsequent susceptibility of broilers to IBD infection.

## 5. Conclusions

This study was conducted to simulate field conditions and evaluate the efficacy of the five IBD vaccination programs in commercial broilers (with high MDAbs), which are administered without determining the optimal time for vaccination by using parameters such as clinical signs, postmortem macroscopic and microscopic lesions, mortality rate, FCR, serology, spleen index, and bursal index. We summarize that the HVT-IBD vector vaccine (group A) at one day old was safer and gave superior protection against the vvIBDV challenge followed by the immune-complex vaccine (group B) at one day old compared to other vaccinated groups. The use of killed IBD vaccine at an earlier age in broilers strengthened the protection induced by the double doses of intermediate vaccines on broilers with high maternally derived antibodies against the vvIBDV challenge. 

## Figures and Tables

**Table 1:** Mortality % and feed conversion rate (FCR) at seven days post challenge with very virulent infectious bursal disease virus (vvIBDV).

Group	Mortality %	FCR ± SD
A	0	1.65 ± 0.15 ^b^
B	0	1.58 ± 0.11 ^b^
C	10	1.80 ± 0.10 ^ab^
D	10	2.25 ± 0.46 ^a^
E	0	2.20 ± 0.56 ^a^
PC	20	2.41 ± 0.42 ^a^
NC	0	1.54 ± 0.11 ^b^

(A): Herpesvirus of Turkey-Infectious Bursal Disease (HVT-IBD) vector vaccine at hatchery. (B): Immune complex at hatchery. (C): Intermediate plus (228E) at 14 days of age by eye drop. (D): Two doses of intermediate (D78) at 12 and 22 days of age by eye drop. (E): Like group D but inoculated also with IBD killed vaccine s/c at 5 days of age. (PC &NC): Positive and negative control birds did not receive any IBD vaccines but challenged at 28 days of age with vvIBDV (PC) and saline (NC) by oculo-nasal route. Different superscript letters indicate significant differences between experimental groups (*p* < 0.05, ANOVA).

**Table 2:** Bursal index, Bursal lesion score, and spleen index at 35 days on challenged and non-challenged birds.

Groups	Bursal Index ± SD		Average Bursal Lesion Score ± SD		Spleen Index ± SD	
	No ch	7 dpch	No ch	7 dpch	No ch	7 dpch
A	0.83 ± 0.06 ^ab^	0.57 ± 0.05	0.00 ± 0.00 ^c^	0.83 ± 0.17 ^c^	0.76 ± 0.15 ^ab^	0.98 ± 0.02 ^ab^
B	0.50 ± 0.08 ^c^	0.35 ± 0.06	1.33 ± 0.17 ^b^	2.00 ± 0.29 ^b^	1.07 ± 0.14 ^a^	0.73 ± 0.20 ^b^
C	0.35 ± 0.06 ^c^	0.33 ± 0.04	1.00 ± 0.00 ^b^	2.17 ± 0.33 ^b^	0.58 ± 0.11 ^b^	0.78 ± 0.10 ^b^
D	0.46 ± 0.04 ^c^	0.34 ± 0.03	0.33 ± 0.33 ^c^	2.00 ± 0.00 ^b^	0.75 ± 0.06 ^ab^	0.56 ± 0.01 ^b^
E	0.60 ± 0.02 ^bc^	0.38 ± 0.09	1.83 ± 0.17 ^a^ (4/6) *	2.00 ± 0.00 ^b^	0.73 ± 0.13 ^ab^	0.80 ± 0.15 ^b^
No vaccine	1.02 ± 0.23 ^a^	0.39 ± 0.02	0.00 ± 0.00 ^c^	3.17 ± 0.17 ^a^	1.01 ± 0.12 ^ab^	1.31 ± 0.32 ^a^

Bursal Index according to [34]. Bursal lesion score as following: 1 = 1–25%, 2 = 26–50%, 3 = 51–75%, 4 = 76–100% of follicles showing lymphocytic depletion and/or necrosis [31]. Spleen index according to [29]. No ch: No challenge. 7 dpch: 7 days post challenge. SD: Standard Deviation. * Four of six birds showed histological lesions on this group and in all groups six out of six birds showed lymphocytic depletion and/or necrosis. Different superscript letters in the same column indicate significant difference between groups (*p* < 0.05).
